# Bioplausible multiscale filtering in retino-cortical processing as a mechanism in perceptual grouping

**DOI:** 10.1007/s40708-017-0072-8

**Published:** 2017-09-08

**Authors:** Nasim Nematzadeh, David M. W. Powers, Trent W. Lewis

**Affiliations:** College of Science and Engineering, Flinders University, GPO Box 2100, Adelaide, SA 5001 Australia

**Keywords:** Visual perception, Cognitive systems, Pattern recognition, Biological neural network, Self-organizing systems, Classical receptive field (CRF) models, Geometrical illusions, Tilt effects, Difference of Gaussians, Perceptual grouping, Gestalt grouping principles

## Abstract

Why does our visual system fail to reconstruct reality, when we look at certain patterns? Where do Geometrical illusions start to emerge in the visual pathway? How far should we take computational models of vision with the same visual ability to detect illusions as we do? This study addresses these questions, by focusing on a specific underlying neural mechanism involved in our visual experiences that affects our final perception. Among many types of visual illusion, ‘Geometrical’ and, in particular, ‘Tilt Illusions’ are rather important, being characterized by misperception of geometric patterns involving lines and tiles in combination with contrasting orientation, size or position. Over the last decade, many new neurophysiological experiments have led to new insights as to how, when and where retinal processing takes place, and the encoding nature of the retinal representation that is sent to the cortex for further processing. Based on these neurobiological discoveries, we provide computer simulation evidence from modelling retinal ganglion cells responses to some complex Tilt Illusions, suggesting that the emergence of tilt in these illusions is partially related to the interaction of multiscale visual processing performed in the retina. The output of our low-level filtering model is presented for several types of Tilt Illusion, predicting that the final tilt percept arises from multiple-scale processing of the Differences of Gaussians and the perceptual interaction of foreground and background elements. The model is a variation of classical receptive field implementation for simple cells in early stages of vision with the scales tuned to the object/texture sizes in the pattern. Our results suggest that this model has a high potential in revealing the underlying mechanism connecting low-level filtering approaches to mid- and high-level explanations such as ‘Anchoring theory’ and ‘Perceptual grouping’.

## Introduction

We investigate here whether computational modelling of vision can provide similar interpretation of visual data to our own experiences, based on simple bioplausible modelling of multiscale retinal cell responses to the visual scene. Our visual perception of the world is the result of multiple levels of visual processing. This starts with multiple levels of visual filtering within the retina and ends in multiple levels of processing in the visual cortex. The bottom-up visual processing gives rise to simple percept or features, but multimodal and top-down information flow leads to more complex concepts, as well as influencing basic perception. In our visual system, fast and accurate visual processing functions as a parsimonious system with minimal redundancy, with the processing in the retina serving different purposes and operating in different ways from the later processing levels of the cortex. We regard this assumption as fundamental to a biologically plausible vision model, and human-competitive computer vision, reflecting our understanding of human vision.

Even given the increasingly detailed biological characterization of both retinal and cortical cells over the last half a century (1960s–2010s), there remains considerable uncertainty, and even some controversy, as to the nature and extent of the encoding of visual information by the retina, and conversely of the subsequent processing and decoding in the cortex (see e.g. the review of physiological retinal findings by Field and Chichilnisky [1] and Golish and Meister [2]).

### History of Geometrical illusions

The visual distortion experiences we encounter in visual illusions give clues as to some of the biological characteristics of our visual processing that result in some erroneous perception. The explanations of optical illusions rely on our interpretation of the world, and the ambiguities against our visual experiences result in the illusory percept.

There are various types of optical illusions, and in "Appendix" (Table 1) we illustrate important representatives of the various families including impossible 3D arrangements such as Penrose Triangle and Penrose staircase [3–5], stimuli with multistable perception, flipping back and forth between different perception such as Necker cube [6]; also Herring’s and Orbison’s illusions [7–9] consist of horizontal and vertical lines located on a part of a radial display, inducing tilt/bow/bulge as a result of the three-dimensional percept and the perspective clues. Hermann Grid and Mach Bands have commonly accepted explanations involving the low-level visual retinal/cortical processing by simple cells [10, 11] and the Lateral Inhibition (LI) mechanism, which some of them need high-level explanations. Table 1 in “Appendix” reflects similarity of illusions, but due to the shortage of space and table arrangement, it may not exactly match other illusion classifications [12] based on other explanations. A complete reference list to the source and original illusory patterns given in Table 1 is also provided in “Appendix” (Table 2). A neurobiological explanation for a variety of Geometrical illusion can be found in [13].

**Table 1:**
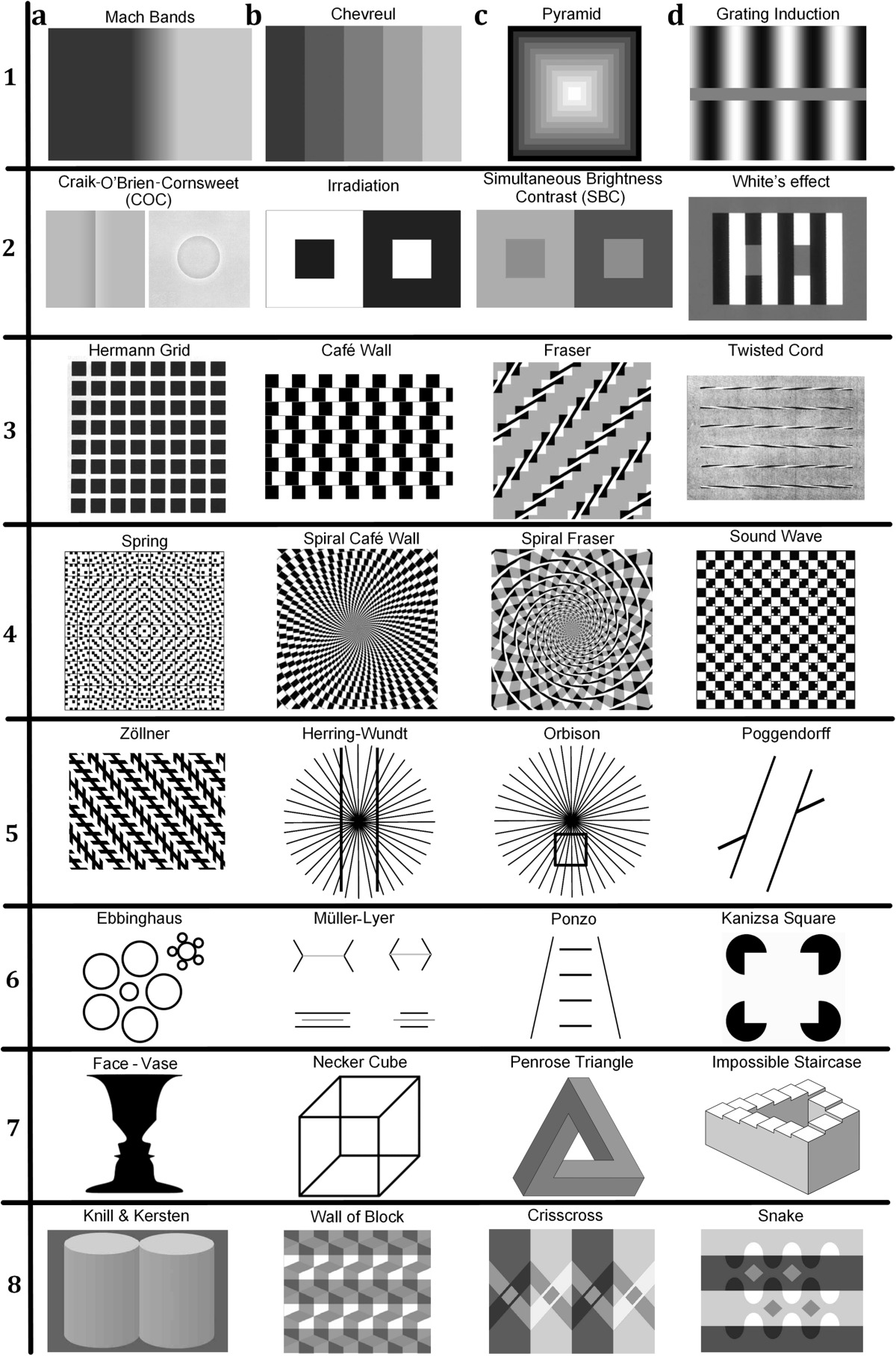
Geometrical and Brightness/Lightness Illusion patterns (Reproduced by permission from [145]). The source and original references of illusions in this table are provided in Table 2

**Table 2:** The source and original references of the illusion patterns in Table 1

Illusion patterns	Appendix Row#-a…d	Source/original Author (date)	References Source/original
Mach Bands	Rl-a	Penacchio et al. (2013)/Mach (1865), Fiorentini (1972)	[63]/[146, 147]
Chevruell	Rl-b	Geier (2011)/Chevreul (1890)	[148]/[149]
Pyramid	Rl-c	Troncoso et al. (2005)/Vasarely (1966, 1970)	[150]/[151, 152]
Grating Induction (GI)	Rl-d	Penacchio et al. (2013)/McCourt (1982)	[63]/[153]
Craik–O’Brien Cornsweet (COC)	R2-a	Web Img, Lu & Sperling (1995)/Craik (1940), O’Brien (1958), Cornsweet (1970)	[154, 155]/[156–158]
Irradiation	R2-b	Westheimer (2007)/Helmholtz (1896)	[45]/[62]
Simultaneous Brightness Contrast (SBC)	R2-c	Adelson (2000)/Heinemann (1955)	[19]/[159]
White’s effect (WE)	R2-d	Blakeslee and McCourt (1999)/White (1979)	[17]/[15]
Herman Grid	R3-a	Blakeslee and McCourt (1997)/Hermann (1870)	[106]/[160]
Café Wall	R3-b	Nematzadeh (2015)/Gregory (1973), Munsterberg (1897), Pierce (1898), Fraser (1908)	[50]/[161–163, 33]
Fraser	R3-c	Kitaoka (2007)/Fraser (1908)	[71]/[33]
Twisted cord	R3-d	McCourt (1983)/Fraser (1908)	[41]/[33]
Spring	R4-a	Fermuller & Malm (2004)/Kitaoka (2003)	[107]/[164]
Spiral Café Wall	R4-b	Kitaoka (2007)/Kitaoka et al. (2001)	[71]/[165]
Spiral Fraser	R4-c	Kitaoka (2007)/Gregory & Heard (1979), Kitaoka et al. (2001)	[71]/[42, 165]
Sound Wave	R4-d	Stevanov et al. (2012)/Kitaoka (1998)	[166]/[109]
Zöllner	R5-a	Web–Wikipedia/Zöllner (1862)	[167]/[168]
Herring–Wundt	R5-b	Changizi et al. (2008)/Herring (1861), Wundt (1898)	[9]/[169, 170]
Orbison	R5-c	Changizi et al. (2008)/Orbison (1939)	[9]/[171]
Poggendorff	R5-d	Ninio (2014)/Zöllner (1860)	[8]/[172]
Ebbinghaus	R6-a	Ninio (2014)/Ebinghaus (1902)	[8]/[173]
Müller–Lyer	R6-b	Ninio (2014)/Müller-Lyer (1889)	[8]/[174]
Ponzo	R6-c	Ninio (2014)/Ponzo (1910)	[8]/[175]
Kanizsa Triangle	R6-d	Eagleman (2001)/Kanizsa (1974), Frisby & Clatworthy (1975)	[13]/[176, 177]
Face–Vase	R7-a	Sturmberg (2011)/Rubin (1915)	[178]/[179]
Necker Cube	R7-b	Sturmberg (2011)/Necker (1832)	[178]/[180]
Penrose Triangle	R7-c	Web—OpenClipArt/Pappas (1989)	[181]/[182]
Impossible Staircase	R7-d	Web—Wikipedia/Penrose (1958)	[183]/[184]
Knill & Kersten	R8-a	Adelson (2000)/Knill & Kresten (1991)	[19]/[185]
Wall of Block	R8-b	Logvinenko et al. (2002)/Adelson (1993)	[21]/[186]
Crisscross	R8-c	Adelson (2000)	[19]
Snake	R8-d	Adelson (2000)	[19]

The patterns explored in this paper are ‘second-order Tilt’ Illusions [14] (Tile Illusions) involving the enhancement of contrast between textural elements of a background such as a checkerboard, for example Café Wall and Bulging checkerboard illusions. In the Café Wall illusion, the illusory tilt percept is the result of mortar lines between shifted rows of black and white tiles. The mortar lines have an intermediate brightness between the brightness of tiles, giving rise to appearance of mortar lines as divergent and convergent instead of parallel lines. On the other hand, in the Bulge patterns, superimposed dots on top of a simple checkerboard give rise to the impression of a bulge or tilt. This is highly affected by the precise position of dots. The illusory perception of these patterns is connected to the figure and ground perception, and in particular, in the Bulge patterns, grouping of dots together creates an illusory figure shape, on top of a textured background (here a checkerboard can be also a grid). This produces apparent border shifts of the checkerboard edges and increases or reduces the impression of the bulges or bows in these patterns.

### Simultaneous Brightness/Lightness Illusions

To be able to differentiate between competing explanations for Geometrical illusions, consider the existing techniques for explaining the simultaneous Brightness/Lightness Illusions such as variations of White’s effect [15–17]. When investigating Brightness/Lightness Illusions, high-level explanatory models are sometimes seen as a result of lightness shift of the same luminance (thus brightness), which decodes as different lightness for example, in [18]. High-level explanations need higher cortical processing of visual clues such as lightness/transparency as well as past experiences and inferences [19–21]. At the same time, to address their final percept, they might involve ideas of interpolation (1D) or filling-in (2D) [22–25] as well. More recently, the ‘Anchoring theory’ of Gilchrist et al. [26] is based on ‘grouping factors’ that signal depth information, without any consideration of the spatial frequency of the pattern. Further explanations for these illusions rely on ‘Junction analysis’ like T-junctions [27] and ‘Scission theory’ [28, 29] which triggers the parsing of targets into multiple layers of reflectance, transparency and illumination in which erroneous decomposition leads to Brightness/Lightness Illusions.

Modern ‘low-level theories’ on Brightness/Lightness Illusions, for example Kingdom and Moulden [30], and Blakeslee and McCourt [17, 31], suggest that a set of spatial frequency filters at early stages of visual processing, mainly preprocessing in the retina are responsible for some Brightness/Lightness Illusions. Recent investigations on diverse range of lightness/brightness/transparency (LBT) illusions by Kingdom [32] have shown different origins for some of these effects due to whether illusion arises from encoding of brightness or the lightness of the pattern. He concluded that the most promising developments in LBT is a model of brightness coding based on ‘multiscale filtering’ (models such as [17]) in conjunction with ‘contrast normalization’.

### Tilt illusions

Throughout the history of Geometrical illusion, a variety of low-level to high-level explanations have been proposed covering many ‘Tilt Illusion’ patterns since Herring’s and Helmholtz time as reflected in the recent overview by Ninio [8]. However, there has been little systematic explanation of model predictions of both illusion magnitude and local tilt direction of tilt patterns that reflect subjective reports from the patterns especially on the chosen Tile Illusions. Although Ninio investigated many Tilt Illusion patterns and presented several principles such as Orthogonal Expansion and Convexity Rule, he stated that these explanations cannot address the family of twisted cord [33] and Tile Illusions (investigated here), even though some of these principles might be part of the explanations. There are also other theories for explaining these illusions. For example, Changizi and his team propose a new empirical regularity for systematization of illusions [9], motivated by the theory of ‘perceiving-the-present’. This theory is based on the neural lag, which is a latency of 100 ms between retinal stimulus and final perception. The well-known hypothesis of ‘perceiving-the-present’ [34, 35] has its foundation in the belief that ‘the visual system possesses mechanisms for compensating neural delay during forward motion’ [9, p. 459] and that we tend to perceive the present rather than perceiving the recent past. Although the hypothesis has been applied for explaining Geometrical illusions in the past [36–39], Changizi’s new prediction generalized this idea and categorized Geometrical illusions based on the central idea that ‘the classical Geometrical illusions are similar in kind to the projections observers often receive in a fixation when moving through the world’ [9, p. 461]. There are some critiques of Changizi’s systematization of illusions,^1^ such as Brisco’s article [40].

However, Tile Illusions have not been explained completely by these so-called generalized theories. Many of these patterns might generally be considered as high-level illusions relying on later cortical processing for their explanation such as Complex Bulge patterns. In the Tile Illusions, the Café Wall illusion [41–47] is the main pattern, which has been investigated broadly. In our previous work [48–50], we have shown that a simple model of multiscale retinal/cortical processing, in the early stages of vision, is able to highlight the emergence of tilt in these patterns with the main focus on the Café Wall pattern.

The current explanation techniques available for investigated patterns are mainly based on three different approaches including the theory of ‘Brightness Contrast and Assimilation’ mentioned by Jameson [51] and highlighted in Smith et al. [52], ‘Perceptual inferences and Junctions analysis’ providing high-level explanations (Grossberg and Todorovic [23]; Gilchrist et al. [26]; Anderson and Winawer [29]) and ‘Low-level spatial filtering’ (Morgan and Moulden [46]; Earle and Maskell [44]; Arai [53]). It is often not obvious how substantive the difference between the explanations is, or whether these explanations are just combinations of orthogonal mechanisms or compatible theories. For example, quite recently, Dixon et al. [54] combined the ‘Low-level filtering’ explanation of Blakeslee and McCourt [17, 31] with higher-level models such as ‘Anchoring Theory’ [26], observing that the key idea or common principle in multiscale, inference base and Brightness/Lightness perception is ‘high-pass filtering tuned to the object size’.

Based on new biological insights, it is now clear that the retinal output is a stack of multiscale outputs (more details in Sect. 3) and modelling this multilayer representation has a significant power in revealing the underlying structure of the percept in computer vision (CV) models [55–59] such as edges, shades, some textures and even some preliminary cues about the depth information, according to some neurocomputational eye models such as [60, 61]. We adopt a parsimonious approach to modelling vision, in terms of both organizations of complexity and computational cost.

### The focus of our investigation

**Fig. 1:**
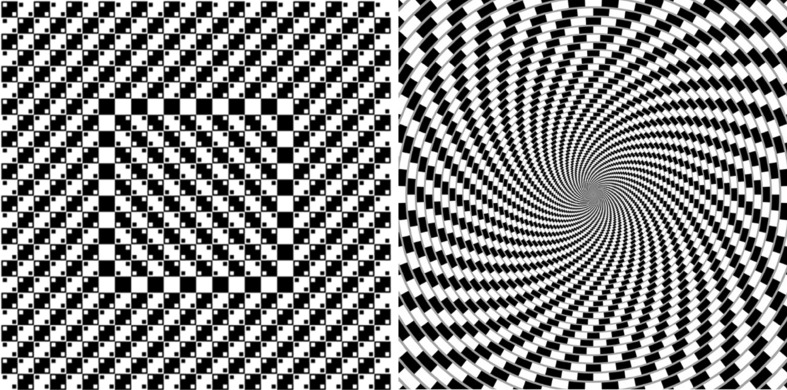
Sample Tile Illusion patterns. *Left* Trampoline pattern [70], *right* Spiral Café Wall illusion [71]

The patterns investigated have some similarities to Brightness/Lightness Illusions such as Irradiation [45, 62], Simultaneous Brightness Contrast (SBC) [63–65] and White’s effect [15, 63, 65–69], but the difference between the explanation of these two subclasses of illusion is that for Tilt Illusions we seek for the prediction of tilt not the changes of Brightness/Lightness profile used to describe Brightness induction effects. The patterns under investigation seem to have mid- to high-level perceptual explanations, but in our previous investigations [14, 48–50] we have shown that the illusory tilt cues in these patterns are caused by multiscale retinal/cortical encoding by the simple cells and the Lateral Inhibition among them. We demonstrated how a low-level explanation of these patterns at multiple scales reveals the tilt cues in the local processing of the pattern, followed by higher levels of processing in the retina and the cortex for the integration of the local tilt cues for the final percept. Two samples of Tile Illusions are shown in Fig. 1, namely Trampoline [70] and Spiral Café Wall [71] patterns.

In this study, we further explore the neurophysiological model of multiple-scale low-level filtering developed by Nematzadeh et al. [14], based on the circular centre and surround mechanism of classical receptive field (CRF) in the retina. For filtering, a set of the Differences of Gaussians (DoGs) at multiple scales is used to model the multiscale retinal ganglion cell (RGC) responses to the stimulus. The simulation output is an edge map representation at multiple scales for the visual scene/pattern, which utilized to highlight the tilt effects in the investigated patterns here. A systematic prediction of perceptual tilt is presented in [48–50] using Hough space [72] for quantitative measurement of tilt inside the DoG edge map. This multiple-scale representation has some analogy to Marr’s and Hildreth [73] suggestion of retinal ‘signatures’ of the three-dimensional structure from a raw primal sketch, this being supported by physiological evidence [1, 74, 75].

One connection of our model with existing explanations is the concept of assimilation and contrast in perceived brightness. Jameson’s dual model of ‘Brightness Contrast and Assimilation’ [51] explains Brightness/Lightness Illusions in terms of DoG filters with different characteristics and dimensions. Here, the ratio of the filter size to image features results in some brightness shifts, contrast or assimilation. Also this filtering representation at multiple scales might be the underlying mechanism to connect similar explanations such as ours with some mid- to high-level explanations for example ‘Anchoring theory’ [26] and its extensions such as ‘Double-Anchoring’ [76] and the idea of illumination framework proposed to address brightness induction effects. Another important outcome of this DoG edge map representation is that it highlights a possible neural mechanism in ‘perceptual organizations’ for local and global percept, the idea in Gestalt psychology for perceptual grouping of pattern elements. What we mean by ‘pattern elements’ are smaller elementary components of patterns that lead to the final percept in general and perceiving illusions in particular.

In the next section, we present the psychological view of perceptual grouping and Gestalt psychology in visual perception. The aim is to bridge between low-level spatial frequency filtering (mainly retinal preprocessing) and high-level perceptual organization (Sect. 2). We then move to a detailed examination of the role of multiscale representation in computational models of vision, with a focus on evidence of multiscale filtering within the retina (Sect. 3) contrastively with other models and theories available in prediction of Brightness/Lightness Illusions and Tilt Illusions. Next (Sect. 4), we explain the details of our simple bioplausible Difference of Gaussian (DoG) model, implementing a classical receptive field (CRF) of simple cells in the retina/cortex, in which their scales are tuned to the object size, ending with experimental results. We conclude by highlighting the outcomes, advantages and disadvantages of our simple visual model and proceed to outline a roadmap of our future work (Sect. 5).

## Perceptual grouping

The perceptual view of visual psychology is mainly based on Gestalt psychological findings [77–79]. These are related to the laws about perceiving meaningfully and generating whole forms by the brain as a global figure rather than recognition of its simpler elements such as lines and points. Therefore, the outlook of Gestaltism in perception is conceptually different from the structuralist view and hence criticized by some scholars from computational neuroscience and cognitive psychology. They claim that Gestalt principles can just provide descriptive laws rather than a perceptual processing model [80]. However, the idea of Gestalt psychology attracted many scholars on relevant areas of vision research, which led to many research findings on object recognition and pattern perception in general [81].

Two major reviews of empirical and theoretical contributions of Gestalt psychology in visual perception by Wagemans et al. and Spillmann [77–79] have been released to clarify the meaning and importance of this concept and to bring it under the attention of researchers in the field of vision. The book ‘Visual Perception’ [79] is among a few that aimed primarily to correlate perceptual phenomena to their underlying neural mechanism. Modern NeoGestaltist views on cortical processing relates to how the brain is working. ‘Often the whole is grasped even before the individual parts enter consciousness’ [82, p. 10]. This ‘arises from continuous global process in the brain, rather than combinations of elementary excitations’ [82, p. 11].

There are a few well-known visual theories in the same spirit as Gestalt. One is the ‘Reverse Hierarchy theory’ [83] claiming that there is a fast feedforward swap that quickly activates global percept in high-level areas with large receptive fields (RFs). There is feedback from these higher areas to lower areas and recurrent processing in the lower-level areas. Processing in lower areas is with small receptive fields for fine-grained processing of local detail of visual input. Another theory is ‘General Theory of Visual Object Recognition’ [84], in which two streams of processing occur in parallel: high spatial frequencies of input images which are processed relatively slow, and in a feedforward sequence in the visual cortex (V1, V2, V4 and so forth), and, on the other hand, low special frequencies of visual input, which are quickly transmitted to area in prefrontal cortex to identify the object as well as their most likely scene context. These two streams are integrated and refined in an interactive, reiterant way for the final percept. Both of these theories postulate that global processing comes first, and they have both dynamic views on cortical processing.

In this context, Spillmann et al. state that ‘Over the years, theoretical accounts for RF properties have progressively shifted from classic bottom-up processing towards contextual processing with top-down and horizontal modulation contributing. These later effects provide evidence for long-range interaction between neurons relevant to figure–ground segregation and pup out by brightness, color, orientation, texture, motion, and depth’ [85, p. 1]. Specific models for uniform surfaces, filling-in and grouping [86, 87] have been formulated and tested to enable the transition from local to global processing by using information from the beyond the classical RFs [85]. It has also been shown that Gestalt factor of good continuation is critical for contour integration [88].

It has been proven that neuronal response not only depends on local stimulus analysis within the classical RFs, but also from global feature integration as a contextual influence, in which it can extend over relatively large regions of the visual field [89]. This is another evidence for the Gestalt credo that a whole is not reducible to the sum of its parts [85]. ‘Classical RFs increase in size from near foveal to peripheral location, from V1 to higher areas in the extrastriate cortex. Smallest in the primary visual cortex (V1), larger in V2, larger again in V3A and V4. Also the slope of the functions describing the increase in size with eccentricity increase progressively from lower to higher visual area’ [85, p. 7].

The Gestalt principles of object and element perception are about grouping of objects based on their similarity, proximity or other cues. Within this global perception processing, there are some innate mental laws reviewed in Wagemans et al. [78]. We believe that in general, Gestalt grouping laws of ‘closure’, ‘proximity’, ‘similarity’ and ‘continuity’ are among the principles, which their underlying neural mechanism can be revealed by some extent by low-level vision models. The simple implementation of retinal/cortical multiscale encoding in our model [14, 48–50] provides some basic understandings of these Gestalt principles, which will be explained further for investigated patterns in Sect. 4.2.

The aim here is to connect multilevel explanations from perceptual organization with global and local percept, to the background low-level filtering explanations. There is a similar connection of low-level retinal/cortical processing to high-level Gestalt grouping principles stated by other researchers in the field. For instance, Craft et al. [90] propose a neural mechanism of ‘figure–ground organization’ based on border ownership to model complex cells in the primary visual cortex (V1). In their investigation of grouping cell connections, they linked their findings to Gestalt grouping principles such as ‘connectivity’, and ‘convexity’ by applying connection weights based on different sizes of receptive fields in the cortex which was modelled based on multiscale and orientated DoG filters. Similarly, Roelfsema’s [91] findings in the perception of ‘pathfinder’ suggest that the Gestalt principle of ‘good continuation’ can be understood in terms of the anatomical and functional structure of the visual cortex.

We have shown [14, 48–50] that the retinal/cortical simple cells processing, simulated in this model for these stimuli (Tile Illusions), explains the emergence of tilt in these patterns. It also provides a basic connection to perceptual grouping of pattern elements and their relational organizations by simple modelling of classical receptive fields (CRFs), tuned to the object size. We will show in Sect. 4.2 how a specific group of pattern elements can be generated in the edge map based on different parameters of our bioderived DoG-based model, simulating the responses of simple cells in the retina/cortex to a stimulus.

We close this section with the following key points by Wagemans who asserts that ‘True Gestalts are dynamic structures in experience and determine what will be wholes and parts, figure and background’ [82, p. 18]. ‘In fact, Gestalt phenomena are still not very well integrated into mainstream thinking about the visual system operating principles (e.g. selectivity and tuning of single cells, V1 as a bank of filters or channels, increasing receptive field size and invariance at higher levels of the hierarchy)’ [82, p. 18].

## Multiscale retinal/cortical representation/biology and modelling

Hubel and Wiesel [92] showed that our visual system has a multiscale and orientation filtering mechanism referred to as an orientation tuner with columnar representation of cortical cells. In contrast, other classical investigations such as Barlow [93] and Kuffler [94] suggested that the image encoding in the retina is based on a centre–surround organization mechanism in retinal successive layers also known in cortex.

Although physiological evidence of the existence of multiscale filtering in the receptive fields of the retina is well known, we highlight the changing size of Ganglion cells (GCs) with eccentricity (that is their distance from the fovea). A recent biological study [1] indicates there are at least 17 distinct GCs in the retina. Each type has a diverse range of size in relation to the eccentricity of neurones and the distance from the fovea. The layer ganglia, covering the retina, thus include different sizes and scale receptive neurons, resulting in a multiscale retinal encoding of the visual input that will be sent to the cortex. This is our assumption for low-level visual processing in the retina. There are other factors strengthening this assumption, such as ‘Fixational eye movements’ [75] which are critical to prevent fading the whole visual world. So the eyes are never still and even when we are fixating, there is a visual mechanism of continuously shifting retinal image on the retina by factor of a few 10–100 s GCs due to the type of fixational eye movements. These include microsaccades, tremors and drifts, which are unconscious source of eye movements. There are also conscious eye movements such as saccades that result on different retinal cells sensations. All the above-mentioned evidence indicates the multiscale filtering representation in the retina due to the eccentricity relation of the GCs sizes, which is their distance from the fovea.

Beyond the multiscale nature of retinal representation, it has also been shown that some retinal receptive fields have far extended surround seen in the lateral mechanism of both horizontals and amacrine cells in the retina compared to the retinal classical receptive fields (CRFs) size. The idea of non-classical receptive fields (nCRFs) was first introduced by Passaglia et al. [95] with modelling RFs based on widely spread surround. This representation provides some evidence for even specific orientation tuning cells in the retina. Therefore, retinal visual output contains not only the edge map with multiscale edge information including the shades and brightness profile around the edges, but also their orientations as a result of multiscale and orientation processing of some retinal cells. This along with other evidence suggests that in many stages of our visual processing, there is spatial filtering adjustment involved such as eye movements, which creates an adaptable spatial size mechanism in the retinal ganglion cells [54]. Shapiro and Lu [64] argue that for brightness perception, the spatial filter size is relative to the object size in the whole image, which is consistent with the spatial vision literature not the retinal spatial frequency. Our DoG model has the same property.

The implementation of the receptive fields (RFs) in both the retina and the cortex based on the Differences of Gaussians (DoGs) dates back to 1960s when Rodieck and Stone [96] and Enroth-Cugell and Robson [97] showed an efficient model for retinal ganglion cell responses and the centre–surround antagonistic effect. The explanations for some Geometrical illusions such as Mach Bands, SBC and Hermann Grid rely on the ‘Lateral Inhibition’ and ‘contrast sensitivity’ of the RGCs [98, 99]. A model of ontogenesis of lateral interaction functions was derived mathematically by Powers [100] showing theoretically that it was possible for commonly assumed types of lateral interaction function to self-organize and in particular approximate the DoG and LoG models, as well as other models related to the Poisson and Gaussian distributions. Considering how this bootstraps to a higher-level model, such distributions can then explain the repeated patterns of edge detectors at particular angles through a self-organizing model [101]. Our investigation is based on applying a low-level DoG filtering model, simulating the responses of RGCs to the Tile Illusion stimulus, to find a lateral inhibitory explanation for these illusions.

Despite the controversy regarding low-level explanations for simultaneous Brightness/Lightness Illusions for example [98, 102–104], low-level filtering techniques showed their great power in addressing illusions of type Brightness/Lightness inductions for example [31, 67, 105, 106]. Some of these models build up their methods on nCRFs implementation such as [17, 68] by using elongated surround for orientation selectivity of some retinal RF or cortical cells. Tile Illusions may have some similarities to Brightness/Lightness Illusions, but they are usually given different explanations [42, 45, 107–110]. We clarify that for Tilt Illusions we should predict and measure the tilt orientation not the brightness profile changes, which is needed for brightness induction explanations, although analysis of brightness might provide us further clues having indirect effect on tilt.

In our previous investigations [48–50], we have shown that a simple classical receptive field model, implementing multiscale responses of a symmetrical ON-centre and OFF-surround RGCs, can easily reveal the emergence of tilt in these patterns. In the future, we intend to extend the model to nonlinear spatial subunits and/or a nCRF implementation in order to identify angles of orientation on detected tilts in the edge map quantitatively instead of our Hough analysis stage.

The novelty of this work arises from its simplicity and the multiple-scale representation view, to explain the emergence of tilt in Tile Illusions based on a DoG edge map of the visual stimulus, in which the scales of filters are determined based on the characteristics of the investigated pattern. More importantly, we believe that the edge map explanation for the tilt effect and visual perception in general bridges the low-level multiscale filtering with high-level explanations of Gestalt perceptual grouping structures by principles such as good continuation and connectivity of the pattern elements. Furthermore, we should state here that gradual changes of DoG scales in here make the model more biologically plausible, and abrupt changes like doubling the scale in each level might end up losing some important information, for modelling CV tools in general and addressing Tilt Illusions.

## Our model

Biological evidence has shown that GC excitation has a centre–surround organization [111] and could be modelled by the differences of two Gaussians [112]. Marr and Hildreth [73] suggest for an involvement of higher-order Gaussian derivatives, utilizing the Laplacian of Gaussian (LoG) and its DoG approximation to model the initial retinal filtering. Young [113, 114] applied linear combination of Gaussians and LoG instead of DoG, but there is still no biological evidence for the structure of these functions [115].

A neurophysiological inspired model implementing the lateral inhibition by the retinal cells [96, 97, 116] is employed by Nematzadeh et al. [14, 48] based on the DoG filtering at multiple scales simulating retinal RF responses to address Tilt Illusions in general. The output of the model is a DoG edge map at multiple scales, in which each scale of the DoG creates a new layer of visual information. Our main intention here is to connect the edge map representation of our model (simulating retinal GC responses to stimuli) to higher-level perceptual grouping mechanisms such as Gestalt grouping principles.

### Multiscale implementation of Difference of Gaussians

The interaction of lateral inhibition by retinal GCs can be modelled by differences of two Gaussians, one for the centre and one for the surround, in which the surround has a larger standard deviation compared to the centre. The DoG filtering techniques are generally used for identifying the edges in CV applications, and by additional multiple-scale DoG filtering, an edge map representation for the pattern is extracted. The multiscale Gaussian representations used in diverse range of CV applications such as DoG/LoG pyramids [58], SURF [117, 118] and SIFT [119] for image representation design, by considering the trade-off between efficiency and complexity for both rendering smooth regions and detailed contours and textures in the image [59]. Due to the bioplausibility goal, we have considered just a simple implementation of multiple-scale DoG filtering and have not used any of those above-mentioned CV techniques to prevent any alternation of the results. Also we change the DoG scales as incremental/decremental stepwise in each level, to capture the maximum output results as the levels of edge map, instead of multiplying the scale in each step, which is mainly used in the CV models.

For a pattern *I*, the DoG output of a retinal GCs model with circular centre and surround organization is calculated as follows:

1 $$ {\text{DoG}}_{\sigma , \, s\sigma } (x,y) = I \times 1/2\pi \sigma^{2} \exp [ - (x^{2} + y^{2} )/(2\sigma^{2} )] - I \times 1/2\pi (s\sigma )^{2} \exp [ - (x^{2} + y^{2} )/(2s^{2} \sigma^{2} )] $$

where *DoG* is the convolved DoG result, *x* and *y* indicate the distance from the origin in the horizontal and vertical axes, respectively, *σ* is the sigma or ‘scale’ of the centre Gaussian ($\sigma = \sigma_{c}$) and $s\sigma$ shows the scale of the surround Gaussian ($s\sigma = \sigma_{{}}$). Therefore, *s* factor is referred to as the *Surround ratio* as shown in Eq. (2). In vision models, this factor is sometimes referred to as point spread function of retinal cells [120].

2 $$ s = \sigma_{\text{surround}} /\sigma_{\text{center}} = \sigma_{s} /\sigma_{c} $$

The value of *s* in our model set to 2. Other values, such as 1.6, were tested with negligible difference in the result. Increasing the *s* factor results in more surround suppression effect on the final output, while the local weights are decreasing (due to definition of a normalized Gaussian kernel).

**Fig. 2:**
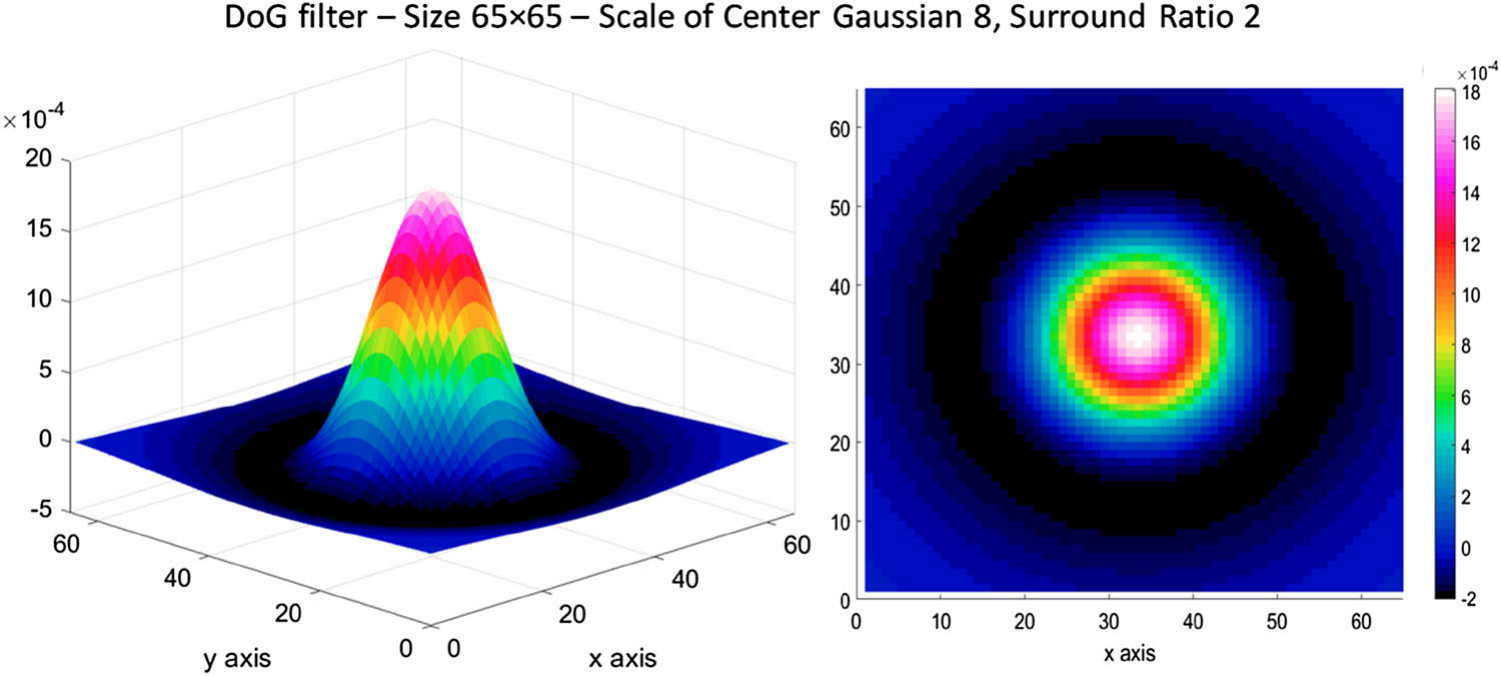
*Left* 3D surface of a Difference of Gaussian filter with the scale of the centre Gaussian equal to 8 ($\sigma_{c} = 8$). The Surround ratio is $s = 2$, and the Window ratio is $h = 8$. *Right* Top view (2D) of the DoG filter. Window size is 65 × 65. Jet white colour map is used to display the graph. (Color figure online)

Rather than the *s* factor for the surround Gaussian, the DoG window size is also another parameter to consider, characterized by parameter *h* given in Eq. (3). Very large windows result in long computation, and very small windows are just approximating a box blur filter not a weighted Gaussian one. For the experimental results (Sect. 4.2), the *h* value set to 8 times larger than the scale of the centre Gaussian ($\sigma_{c}$) to capture the inhibition effect as well as the excitation and keep the significant values of both Gaussians within the window (95% of the surround Gaussian is included within the DoG filter). A three-dimensional surface graph and a top view of a sample DoG filter are shown in Fig. 2 in jet white colour map.^2^

3 $$ {\text{Window size}} = h \times \sigma_{c} + 1 $$

**Fig. 3:**
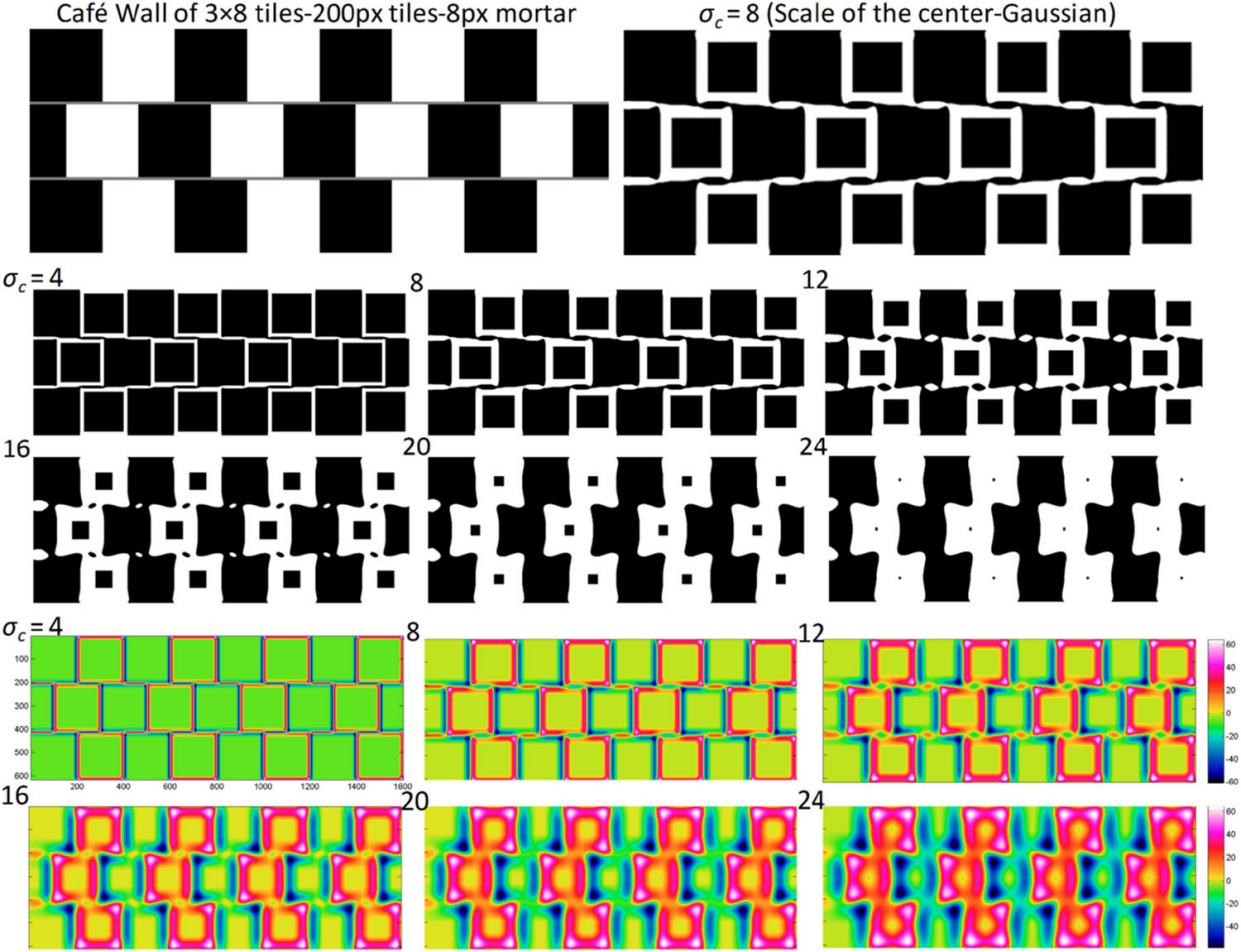
*Top left* Café Wall pattern with 200 × 200 px tiles and 8 px mortar. *Top right* Enlarged DoG output at scale 8 ($\sigma_{c} = 8$) in the edge map. *Centre* The binary edge map at six different scales ($\sigma_{c} = 4\,\,{\text{to}}\,\,24$ with incremental steps of 4). *Bottom* Jet white colour map of the above edge map. Rather than the centre Gaussian, other parameters of the model are: $s = 2$, and $h = 8$ (the Surround and Window ratios, respectively) (Reproduced by permission from [145]). (Color figure online)

**Fig. 4:**
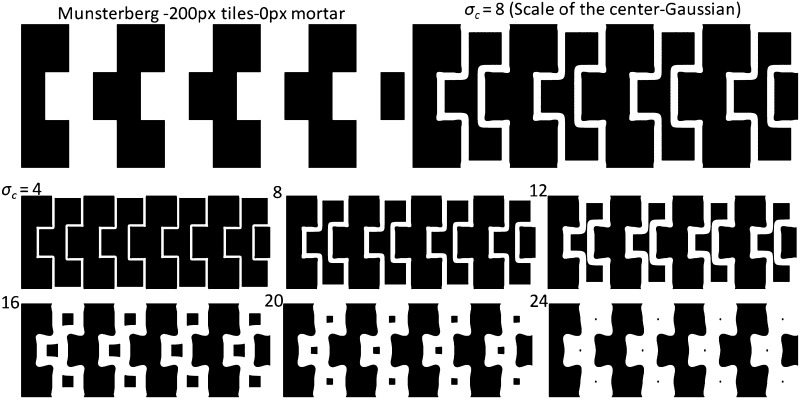
*Top left* Munsterberg pattern with 200 × 200 px tiles. *Top right* Enlarged DoG output at scale 8 ($\sigma_{c} = 8$) in the edge map. *Bottom* The binary edge map at six different scales ($\sigma_{c} = 4\,\,{\text{to}}\,\,24$) with incremental steps of 4. Other parameters of the model are: $s = 2$ and $h = 8$ similar to Fig. 3 (Reproduced by permission from [145])

**Fig. 5:**
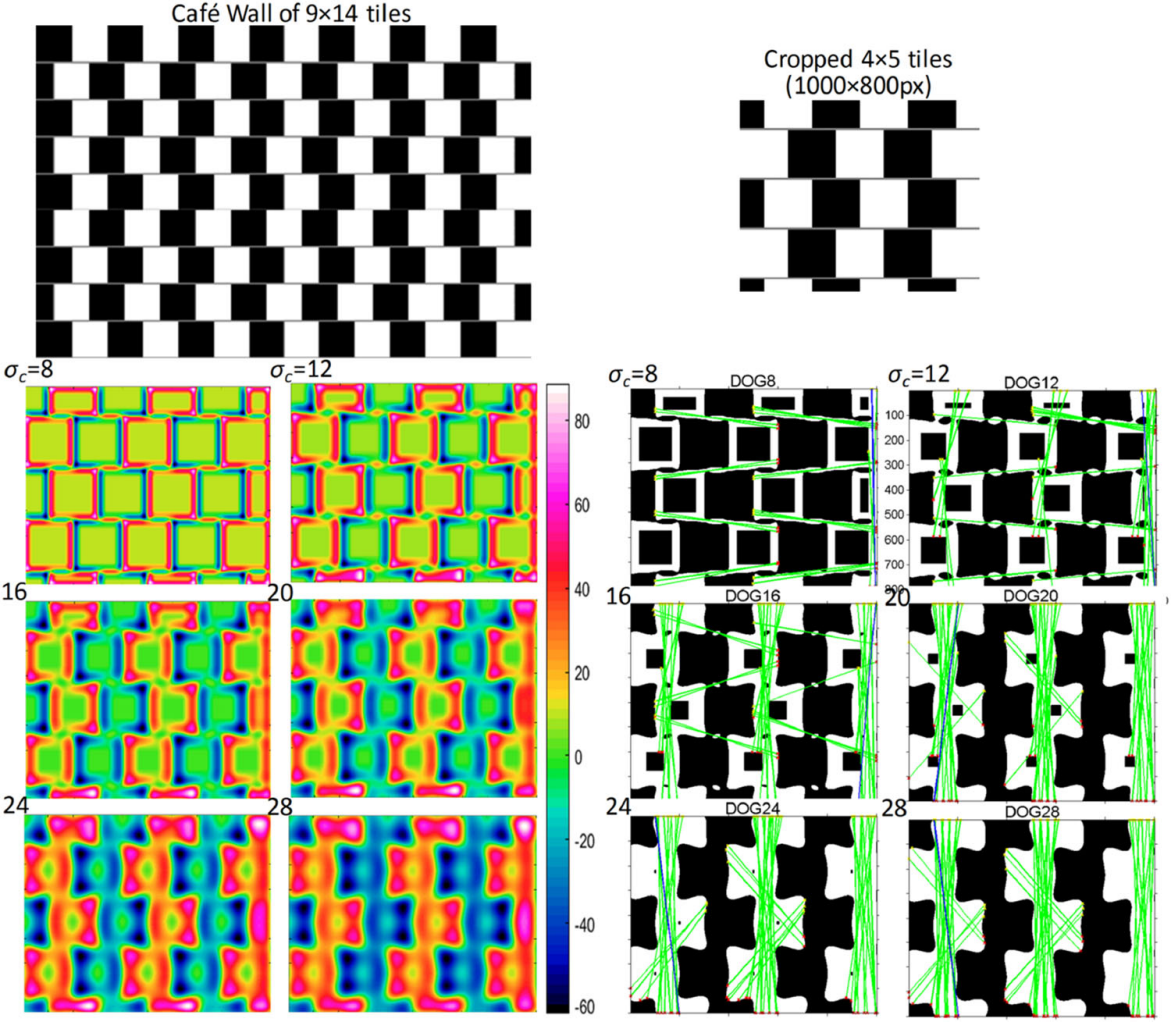
*Top* A crop section of 4 × 5 tiles (enlarged) from a Café Wall of 9 × 14 tiles with 200 × 200 px tiles and 8 px mortar. *Bottom left* Edge map of the crop section at six scales ($\sigma_{c} = 8\,\,{\text{to}}\,\,28$), with incremental steps of 4 in jet white colour map. *Bottom right* Detected houghlines displayed in *green* on the binary edge map and around four reference orientations of *horizontal*, *vertical* and *diagonals*. *Blue lines* indicate the longest detected lines at each scale of the edge map (Reproduced by permission from [145]). (Color figure online)

**Fig. 6:**
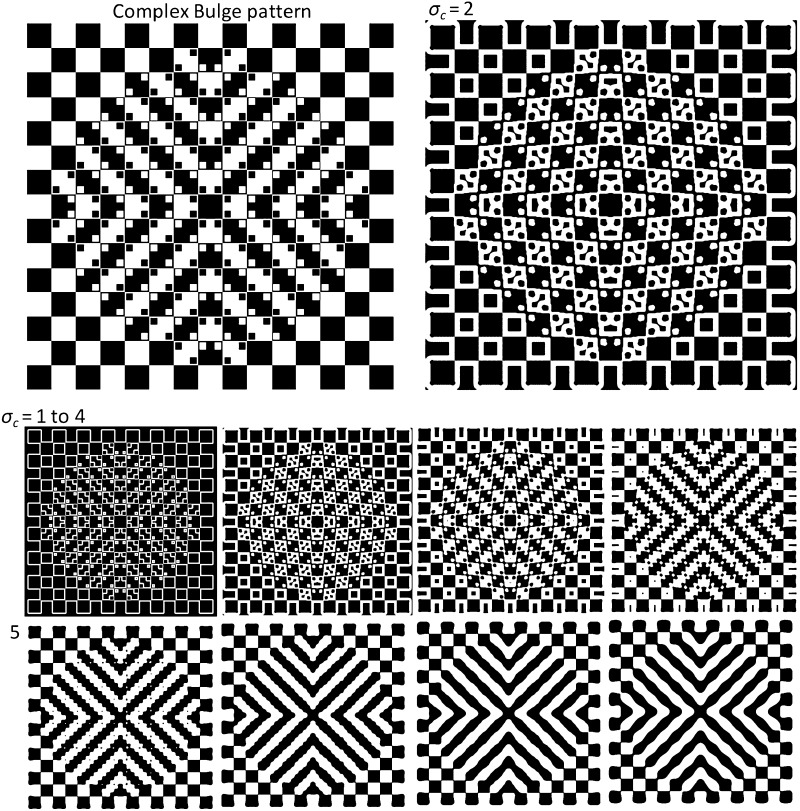
*Top left* Complex Bulge pattern (574 × 572 px) and *top right* The DoG output at scale 2 ($\sigma_{c} = 2$)—enlarged that highlights the bulge effect in the pattern. *Bottom* The binary edge map of the pattern at eight different scales ($\sigma_{c} = 1\,\,{\text{to}}\,\,8$) with incremental steps of 1. Other parameters of the DoG model are: $s = 1.6$, and $h = 8$ (the Surround and Window ratios, respectively) (Reproduced by permission from [145])

So, to generate a multiple-scale DoG representation for the pattern we apply DoG filter at different scales to the pattern. The scales are pattern specific. The outputs of the DoG model, as the edge maps of sample patterns, are displayed in Figs. 3, 4, 5 and 6. On top of these figures, you see the original patterns (Café Wall, Munsterberg, Crop of Café Wall and Complex Bulge patterns) and an enlarged DoG at a specific scale from the edge maps that highlights the tilt effect in these patterns. The edge map representations at multiple scales (may be referred to as multiple-scale DoG edge map for convenience in our work) have been presented at the bottom half of these figures for six or eight scales (six or eight levels of $\sigma_{c}$). Rather than the scales of the centre Gaussian, the other parameters of the model and the characteristics of each pattern have been provided in each figure. In the edge map output, the scale of the centre Gaussian ($\sigma_{c}$) increases first from left to right within a row and then from one row to the next. We tried to represent the multiple-scale representation of our bioplausible low-level filtering model, in a way that the output result can be seen easily as a sequence of increasing scales, which in total provides us the multiple-scale edge map representation for each pattern.

The result of our computational model interestingly reveals the possible underlying neural activation/inhibition effect of the retinal ganglion cell (RGC) responses in our simulations to these illusion stimuli (Tile Illusions). As the results in Figs. 3, 4, 5 and 6 show, the edge map representation of these patterns connect to some ‘Gestalt perceptual grouping’ principles explaining high-level perceptual organization of pattern elements such as ‘proximity’, ‘similarity’, ‘continuity’ and ‘good continuation’. These grouping principles are assumed to be the basic blocks for our perception of the world.

Based on the experimental results provided in Figs. 3, 4, 5 and 6, we found that different grouping structures of pattern elements emerge in the gradual change of scale in the edge map. The grouping structure in the DoG edge map at multiple scales is related to factors such as scale resolution including number of scales and increment/decrement of scale level, the size of DoG filters, as well as the predefined scales in the model which determined by the spatial frequencies of the objects/texture sizes in an investigated pattern which is consistent with spatial vision literatures.

In physiological vision, it is more likely that there is scale adjustment by a preprocessing retinal mechanism or a physical mechanism such as fixational eye movements [75]. This would seem to optimize in some sense for the later retinal processing and the encoding of its multiscale representation for further analysis in the cortex and would involve feedback mechanisms, which provide local supervision in what is overall an unsupervised system, again a matter for further research. This scale adjustment could be modelled in CV by pre-analysis of spatial frequency of the pattern’s features before setting up the scale values for the bioderived DoG model, to produce a parsimonious efficient representation out of the pattern. We have defined the scales of the DoG filters empirically, by considering the size of elements inside each pattern.

Therefore, there is a connection between psychological aspects of a pattern and its structural features (considering the grouping factors of its elements) and a parametric low-level representation of the pattern (as its retinal encoding) implemented in our model. So, by modelling the RGC responses and generating an edge map representation of a pattern, we are able to find a precise given range of DoG scales, which reveals the emergence of a particular perceptual grouping structure from the pattern elements. We will show this further in the following results.

### Experimental results

The output of our bioplausible model [14, 50] for Tile Illusions, including the ‘Café Wall’, ‘Munsterberg’ and Complex Bulge’ patterns, shows that simply utilizing a DoG processing at multiple scales, implementing lateral inhibition in the RGCs, not only produces an edge map when applying a fine-scale DoG, but also extracts other hidden information such as shades and shadows around the edges, and even the local texture information by increasing the scale of the DoG filter.

These results not only highlight the connection of our model to Jameson’s theory of ‘Brightness Contrast and Assimilation’ [51], but also indicate that there are numerous Geometrical cues available that can be uncovered by this simple edge map representation.

There are some previous explanations for connecting ‘Brightness/Lightness’ Illusions and ‘Geometrical’ illusions [46, 52]. The tilt perception in Tile Illusions seems to be affected from ‘Brightness Contrast and Assimilation’ as well as some ‘border shifts’ [14, 45].

The multiple-scale DoG edge map for the ‘Café Wall’ illusion indicates the appearance/emergence of divergence and convergence of the mortar lines in the pattern shown in Fig. 3, similar to how it is perceived. The Café Wall illusion originates from the twisted cord [33] elements with the local inducing tilt and then integration of these local tilts to an extended continuous contour along the whole mortar line [47, 121]. The investigated pattern is a Café Wall of 3 × 8 tiles with 200 × 200 px tiles and 8 px mortar. We start at finest scale below the mortar size at scale 4 ($\sigma_{c} = 4$) and extend the scales till scale 24 ($\sigma_{c} = 24$) here, with incremental steps of 4. At scale 28 ($\sigma_{c} = 28$) which investigated but has not been shown in the figure, the filter captures nearly the whole tiles of the pattern [refer to Eq. (3)]. Non-critical parameters of the model are *s* = 2 for the *Surround ratio* and *h* = 8 for the *Window ratio.* Similar explanations for the Café Wall illusion have been given by other scholars based on either ‘Low-level filtering’ models [41, 43, 44, 46, 110] or ‘higher-level’ psychological explanations such as ‘Border Locking’ explanation [42]. The illusion percept in the pattern could be affected by the intermediate brightness profile of the mortar lines with respect to the brightness of the tiles, the height of the mortar lines, the ratio of the mortar height to the tile size, the amount of tile shifts in consecutive rows (phase of tile displacement) [41, 42] and even more involvement of other perceptual parameters in the pattern. The simulation results of our investigations on variations of Café Wall patterns having different characteristics based on the above-mentioned parameters have been presented in our article [122]. The paper presents both qualitative and quantitative comparison results of the tilt prediction of the model for both the magnitude and the direction of tilt in these variations. We note that even the strength of illusion in different variations of the Café Wall pattern is predictable from the DoG edge map representation at multiple scales. We are not aware of any other theories which have produced quantitative predictions for this illusion.

A closer look at the multiple-scale representation of the Café Wall pattern in Fig. 3 reveals further cues about how perceptual grouping structures are generated during the DoG processing. The grouping of white tiles in two adjacent rows by the mortar line connecting them starts to appear at fine scales at 4 and 8 ($\sigma_{c} = 4,\,8$). As the scale increases, this grouping structure (mortar line connection with the tiles), referred to as ‘twisted cord elements’ [33] of the pattern, starts to get disconnected, as shown at scales 12 and 16 ($\sigma_{c} = 12,\,16$) clearly. At the next scale ($\sigma_{c} = 20$), there is no grouping visible of the tiles and mortar cues, although the appearance of tiles as wedge shape provides a cue to support the near-horizontally diverging and converging perception of the location of the mortar lines, even without the appearance of them in the DoG output at coarse scales. From scale 20 ($\sigma_{c} = 20$) onwards, a new grouping arrangement arises, this time in the vertical direction. This group arrangement connects identically coloured tiles in a vertical zigzag direction exactly in the ‘opposite direction’ to the previously seen (near-horizontal) diverging/converging mortar groupings.

The ‘vertical zigzag groupings’ seem to be the result of our global perception of the pattern, while the near-horizontal diverging/converging’ mortar lines are a local percept arising from a local focus on the mortar lines joining different tiles in adjacent rows. Therefore, there is a simultaneous perception of both groupings due to rapid changes in the focus point of the eye. Even at a focal view, due to eccentricity relation of RGCs sizes and distance to the fovea, we still have this multiscale retinal encoding of the pattern. Although we might get an impression of either the ‘local or global percept’ of the pattern in an instance of time, it is more likely that the global percept would carry more weight in the final perception of the illusion pattern, but interestingly, even at coarse scales, where tiles are not connected through the mortar lines, the wedge shape of tiles provides the same stable direction for the divergence/convergence percept of the mortar lines in the DoG edge map.

The edge map representation of the Café Wall illusion indicates that, at different scales of the DoG, there are ‘incompatible grouping precepts’. In other words, according to ‘continuity’ principle at scale 8 ($\sigma_{c} = 8$), we see a column where the middle element is displaced to the right, while at scale 20 ($\sigma_{c} = 20$) it is displaced to the left. We claim that the existence of different precepts at different scales contributes to the tilt induction in the Café Wall illusion [14, 48].

To further investigate the contribution of the mortar lines on the tilt effect of the Café Wall pattern, we investigated the ‘Munsterberg illusion’ [41, 46, 47], a version of Café Wall pattern without any mortar lines in between rows of tiles, as shown in Fig. 4. The tile sizes are the same as the Café Wall pattern explained before (200 × 200 px) and the parameters of the model have been kept the same as described in Fig. 3. The Munsterberg pattern and the DoG output at scale 8 ($\sigma_{c} = 8$) have been shown on top, followed by the multiple-scale edge map of the pattern at six different scales at the bottom half of the figure.

The edge map of the Munsterberg pattern (Fig. 4) shows that the early grouping of nearly horizontal diverging and converging tilt cues along the mortar lines seen in the Café Wall pattern at fine scales ($\sigma_{c} = 4,\,8,\,12$; Fig. 3) does not occur with the Munsterberg pattern at all. The only grouping of pattern elements revealed in multiple scales of the DoG edge map here is just the vertical zigzag or columnar groupings of identically coloured tiles; therefore and in direct contrast to the ‘Café Wall’ illusion, the ‘Munsterberg’ pattern has NO illusory perception of tilt. The results support previous psychophysical findings [41, 46, 47].

So, the DoG edge map of the ‘Café Wall’ qualitatively reveals the tilt in the pattern. For quantitative analysis of tilt, we have used Hough space to measure the absolute mean tilt angles of the mortar cues in the edge map of the Café Wall pattern at multiple scales [48–50]. Figure 5 shows the tilt analysis results of a crop section of a Café Wall pattern. The crop section consists of a 4 × 5 tiles and selected from a Café Wall of 9 × 14 tiles with 200 × 200 px tiles and 8 px mortar as shown in Fig. 5 (top). At the bottom left, the edge map at six different scales ($\sigma_{c} = 8\,\,{\text{to}}\,\,28$) with incremental steps of 4 is displayed (in jet white colour map). The bottom right shows the detected houghlines in green, displayed on the binary edge map at six different scales and around four reference orientations of horizontal, vertical and diagonals (blue lines indicate the longest lines detected at each scale, and red and yellow crosses show the beginning and end of detected line segments). For further implementation details about the quantitative measurement of the degree of tilt in the pattern and detected houghlines, please refer to [48, 50].

The other investigated pattern here is ‘Complex Bulge’ pattern shown in Fig. 6. The pattern consists of a checkerboard background and superimposed dots on top of the checkerboard. The edge map of the pattern at eight scales has been shown at the bottom half of the figure, starting at scale 1 ($\sigma_{c} = 1$), the finest scale, to scale 8 ($\sigma_{c} = 8$), with incremental steps of 1. The DoG output at scale 2 ($\sigma_{c} = 2$) has been enlarged and displayed on top of the figure, next to the original pattern. Here the *Surround ratio* is chosen as *s* = 1.6 [73], and the *Window ratio* is *h* = 8. As explained in Sect. 4.1, the ‘distributions of spatial scales’ are specified by pattern elements. In Fig. 6, the size of the Complex Bulge pattern is 574 × 572 px. The dimensions of each individual tile and small dot in the pattern are 36 px and 10 px, respectively. Therefore, in order to capture both high-frequency details (superimposed dots) and low-frequency contents (tiles) from the pattern [using Eq. (3) and the constant value of 8 for the *Window ratio*], the range of scale should start with a filter smaller than the dots and extended to a maximum size larger than the tiles in the pattern (*Window size* = 8 × 1 + 1 = 9 < 10 px; the size of dots; and *Window size* = 8 × 8 + 1 = 65 nearly twice as the size of each tile to specify the range of scales between 1 and 8 with incremental steps of 1 in the edge map of this pattern).

The bulge effect might be addressed by ‘high-level’ explanations such as uncertainties in both formation and processing of image features, for instance points and lines [107] or even ‘psychological’ explanations based on categorization of edges due to different intensity arrangements around them [42, 71], but our explanation relies on a ‘low-level multiscale filtering model to address the tilt/bulge effects in these patterns. Based on our assumptions, the ‘Bulge effect’ is happening due to a number of visual clues, for instance the brightness perception of the checkerboard background causing a simple border shift outwards for the white tiles, and expansions in the intersection angles due to Brightness Contrast and Assimilation theory [51]. More importantly, further clues related to local positions of superimposed dots, which may have frequency discharge or emission results in local border tilts or bows [14].

In the ‘Complex Bulge’ pattern (Fig. 6), the output of our DoG model again predicts two distinct groupings. At fine scales from 1 to 3 ($\sigma_{c} = 1\,\,{\text{to}}\,\,3$), there is a grouping of a two-dimensional bulge, starting from the central tile square, expanding towards the outer regions, like a circular movement of a bulge from the centre to its surroundings. The effect produces a kind of edge displacement or tilt of the checkerboard edges with the induction of the bulge. The DoG output at fine scales ($\sigma_{c} = 1\,\,{\text{to}}\,\,3$) in Fig. 6 highlights a grouping of connected superimposed small dots together, which has a close connection to ‘Similarity’, ‘Continuity’ and ‘Connectivity’ in the Gestalt grouping principles. At fine scales of the DoG edge map, the ‘central tile’ plays an important role in the inducing bulge effect. By increasing the scale from 4 to 8 ($\sigma_{c} = 4\,\,{\text{to}}\,\,8$), another grouping structure starts to emerge, out of identically coloured tiles with an X-shape structure combined with a wave-like effect between the intersections of the X. Unlike the first bulge grouping, which occurred as a result of fine-scale DoG filtering, the DoG output of the pattern at coarse scales results in edge movements of the ‘central tile’ with the tilt effect appearing in a different direction (nearly 45° rotated from the grid) due to the ‘X-shape grouping’ structure, which groups the background checkerboard tiles together. Again, ‘Similarity’, ‘Connectivity’ and ‘Continuity’ are the Gestalt grouping principles that have been shown by our simulated results modelling low-level processing of the simple cells in response to the pattern. The difference between the structure of the X-shape group and the Bulge group is that the X-shape grouping occurs for coarse or low-frequency components of the pattern, that is the checkerboard tiles, rather than the high-frequency details, that is the small superimposed dots (appear in the DoGs at fine scales) in which it generates the Bulge group. The X-shape grouping has an inducing effect of shrinkage on the central tile exactly opposite to its previous expansions with the DoG responses at fine scales.

As into the ‘Café Wall’ illusion explained before, in the ‘Complex Bulge’ pattern, the DoG output of the model includes two distinct grouping arrangements, in this case a ‘Central Bulge’ and an ‘X-shape’ induction, with incompatible effects on the border shifts. It seems that the ‘X-shape’ percept arises from global perception when we focus on the periphery of the pattern, while the ‘Bulge effect’ is the result of focusing on the central area close to the central tile or on the inducing superimposed dot cues on the checkerboard; therefore, it is a local percept. To summarize, the multiple-scale DoG edge map of the ‘Complex Bulge’ pattern reveals two distinct and incompatible precepts or grouping structures arising simultaneously and results from our local to global percept of the pattern, contributing to bulge induction in the pattern.

Tilt effect in the Bulge patterns can also be explained in terms of perceptual interaction of foreground and background elements. The checkerboard in these patterns plays as a background and small superimposed dots as the foreground object. Due to the blurring effect of multiscale retinal GCs encoding and multiscale cortical processing of retinal output, these superimposed dots get connected (grouped) together and create a foreground subpattern as an illusory figure. The final perception in these patterns arises from the interaction of foreground superimposed dots (illusory figure of grouped dots) and the background elements of checkerboard tiles. In our implementation, the blurring effect on the final perception of the pattern is related to the size of the DoG filters, which acts as a band-pass filter. It is noteworthy that the shape of dots does not play an important role in the final percept of these patterns, although their sizes and positions (their relative distance to the outlines of the tiles of the checkerboard) in the patterns are important. They can be square, circle or any other shape but as long as their relative sizes are the same, the overall impression of tilt would be similar. The reason is that in the multiple-scale DoG processing, which simulates visual blurring at early stages of visual processing, the outputs get very similar when the size of DoG filter reaches to the overall size of the dots.

**Fig. 7:**
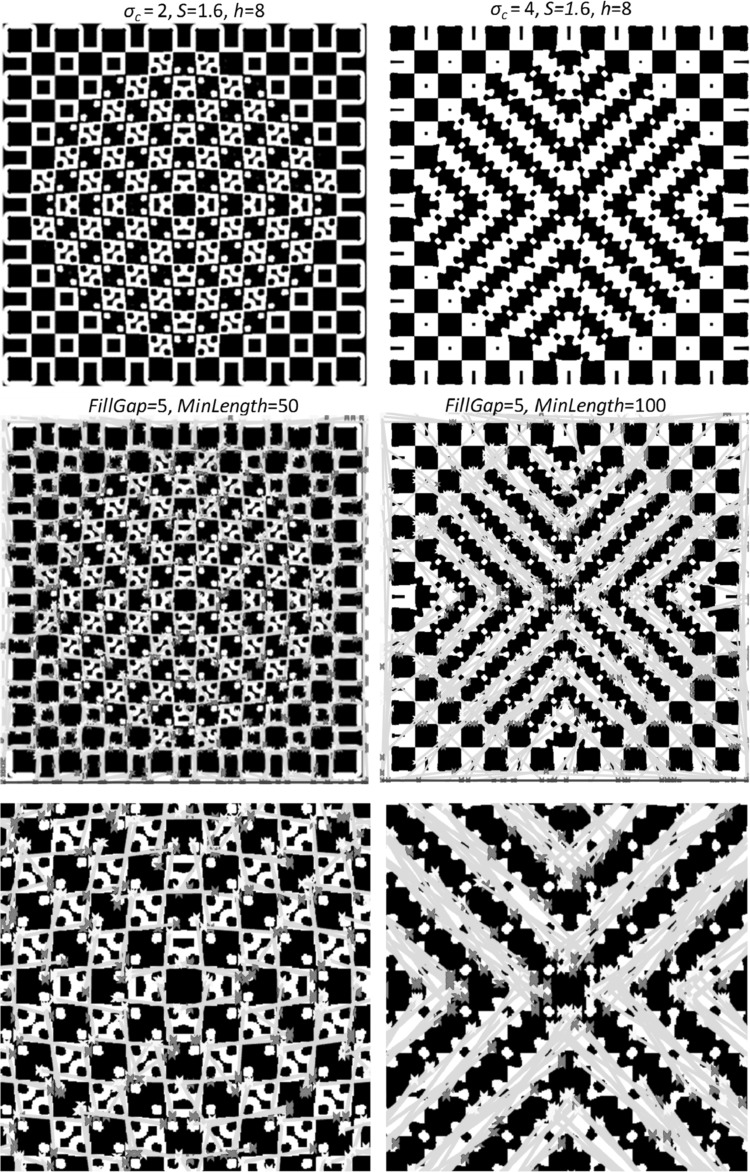
Detected houghlines (*centre*) for two scales of 2 and 4 ($\sigma_{c} = 2,\,4$) from the edge map of the Complex Bulge pattern (*top*). The two images at the *bottom* of the figure are zoomed-in versions of the two images at the *centre row*. The parameters of the model and Hough investigation have been provided on the figure (Reproduced by permission from [145]). (Color figure online)

Rather than just showing the bulge/tilt effect qualitatively in this pattern, we have done some quantitative measurement of tilt in Hough space. Detected houghlines for two scales of 2 and 4 ($\sigma_{c} = 2,\,4$) of the DoG edge map have been displayed in green on the binary edge map in Fig. 7 (centre row). The two images at the bottom row of the figure show the zoomed-in versions of the images in the centre row. Here the technique used for quantitative tilt measurement highlights two different groupings of pattern elements at different scales of the edge map. We have shown this quantitative tilt results and detected houghlines on the Complex Bulge pattern here as a sample to show the coverage of our model for more complex Tile Illusions. Further details about the extraction of houghlines on this pattern are out of the scope of this paper, but for Café Wall pattern it can be found in [48–50].

We should note that although the DoG edge map representation of Tile Illusions reveals the local tilt cues at multiple scales of the edge map (most probably reflects on the encoding of the retinal ganglion cells), these local tilt cues must be integrated at higher levels of visual processing (completed in the cortex). The mechanisms involved in the edge integration are widely believed to be the result of cortical complex cells (see, for example, [47, 110, 121] for the Café Wall illusion). Based on the neurophysiological evidence for the existence of some retinal GCs with orientation selectivity property [93, 123–125], as well as the possibility of a simultaneous activations of a group of GCs (combined activity) in the retina by the output of amacrine cells [126–128], we suggest the possibility that the edge integration starts in the retina to some extent, in which it reflects in the retinal spike trains send to the cortex for the completion of the process.

We are not claiming that our simple multiple-scale DoG model can replace all the mid- to high-level explanations for similar illusions. We believe that many of these approaches have broad value, for example in contour illusion explanations or LBT illusions, which need further analysis or even previous knowledge and inferences for their explanation. What we have presented here builds on previous work [14, 48–50], highlighting the power of a first-stage multiple-scale model (with gradual changes of scale) based on retinal physiological insights and its multiscale processing of a visual scene. The aim was to show how much of visual information can be revealed and encoded by low-level retinal/cortical simple cells processing. This first-stage multiple-scale output can then feed to other high-level models for further application-based processing (for instance reweighting for normalization schemes or by the fusions of multiple scales into a multiscale representation).

Here we have connected the output of our DoG model at multiple scales, to some prominent perceptual grouping principles such as continuity, connectivity and similarity, which are high-level perceptual explanations of our final percept. What we believe is that bioplausible low-level filtering techniques like ‘Lateral Inhibitions’ and ‘Contrast Sensitivity’ models are able to answer many Geometrical and Brightness/Lightness Illusions based on their nature of multiscale processing. We have shown here that simple circular centre and surround implementation of RGCs can reveal the emergence of tilt in the Tile Illusions. Although our model is a classical RF (CRF) implementation of RGC responses at multiple scales, the scales of the DoG edge map have been specified based on the characteristics of the investigated pattern. Additional implementations for orientation tuning of retinal ganglion cells (either nonlinear spatial subunits or nCRF implementations) might facilitate the investigations on broader range of Geometrical and Brightness/Lightness Illusions.

## Conclusions and future work

We investigated the lateral interaction effect in visual processing of the perceptual organization of pattern elements and how it is connected to mid- and high-level result of grouping factors in our global percept, by simple computational modelling of retinal/cortical simple cells. In the perception of a visual scene, the grouping means ‘putting items seen in the visual field together, or organizing image data’ [129, p. 305]. It should be noted that the interest in grouping processes originated with the effort of Gestalt psychologists who have been the first to study grouping comprehensively as part of the general process of perception [130] (1920s, 1930s) and formulated a set of rules to explain the groupings perceived by humans. These grouping principles were typically justified by drawing parallels with certain neurological theories that were known at the time for their underlying neural mechanism.

In computational modelling of vision, Marr pointed out the need for perceptual clustering algorithms for obtaining full primal sketch from the raw primal sketch, using criteria such as collinearity and size similarity [22]. Clustering and segmentation algorithms broadly studied by the researchers in the field of CV and our intention for explaining illusory tilt effect focus on simple modelling of retinal low-level processing. We presented our investigation on a variant of the retinal classical receptive field (CRF) model implementing processing in the retinal ganglia and then used the model to generate an edge map representation as a raw primal sketch, which clearly highlight the emergence of tilt on a few Tile Illusion patterns.

It is well established that the centre–surround receptive fields of the RGCs are ‘contrast estimators’ [97]. We implemented our model based on a circular centre and surround organization of RGC responses considering the lateral inhibition interaction among them and utilized the DoG filtering at multiple scales to explain some of the second-order Tilt Illusions [14, 50] (referred to as Tile Illusions in our study), in particular ‘Café Wall’ and ‘Complex Bulge’ patterns here. We used this DoG edge map at multiple scales on Tile Illusion patterns described here for prediction and measurement of both tilt magnitude and its direction, by further analysis on their DoG edge maps by our model.

Although the edge map representation at multiple scales with gradual change of scale as the model output might seem over-complete, it has the potential to provide a lower error model of the data and is more likely to provide the information at the level of detail required for a particular image or application. One advantage of such models is that the model output is not sensitive to the exact parameter settings. It is important that the range of scales in the bioplausible DoG-based model is defined in such a way as to capture both high-frequency edge and texture details and the low-frequency profile information conveyed by brightness/colour from the objects within the scene. Thus, the neural processing of images that leads to the final potentially illusory output occurs at multiple scale levels. The number of such scales involved is a function of the parameters of the model. In general, reducing the number of scales is possible by increasing the incremental step, but this needs to be managed in a way that does not lose any intermediate or preliminary perceptual information that emerges during visual processing of a pattern or scene.

In this current work, we further investigated this model and the Tile Illusion patterns to find a connection of our ‘bioderived Low-level filtering’ model to some high-level ‘Perceptual grouping’ organizations with our main focus on Gestalt grouping principles, for example continuity (good continuation), connectivity and similarity. The experimental results show that the output of the model as an edge map representation of a stimuli could provide us not only the multiple-scale edge information as the indications for some shades around the edges, but also other hidden information such as local texture in the stimuli, as well as possible underlying mechanism for perceptual grouping arrangements at different scales. Therefore, the implementation of lateral inhibition in RGCs using DoG processing at multiple scales creates a feasible perceptual interpretation of the local structure in the pattern in a way that best meets its global perception.

We expect further that these low-level filtering approaches (retinal/cortical) have the ability to play a significant role in other higher-level models in relation to depth and motion processing which can contribute to the high-level top-down explanations of visual processing and can be extended to nCRF modelling for orientation selectivity of visual complex cells. Even the combination of the nCRF and the CRF for modelling RGCs could go a long way in supporting low-level filtering models. There is still much debate about the extent of coverage of isotropic filters in low-level approaches to represent the visual cues. Two successful models demonstrating the power of symmetrical filters in brightness perception models can be found in [131, 132]. Dakin and Bex [131] proposed a model for amplification of the low spatial frequency information of the image. Their model relies on a reconstruction phase based on the natural statistics of the image using a series of centre–surround, Laplacian of Gaussian filters. They demonstrated that complex models of orientation selective filters are not essential to successfully model brightness phenomenon such as White’s effect and Craik–O’Brien–Cornsweet (COC) illusions. They highlighted the importance of normalization within low-level models rather than elongated filters. Zeman et al. [132] utilized a family of exponential filters (again isotropic) with multiple sizes and shapes instead of DoG/LoG for predicting the perceived brightness in their model. Their simulation results are comparable with the results achieved by the state-of-the-art Brightness/Lightness models [17, 67, 68] addressing both assimilation and contrast effects in Brightness/Lightness Illusions. All of these CV models certainly should get support from physiological evidence of retinal and cortical visual processing.

The extent of retinal encoding of visual input is not yet clear. The complexity of retinal processing of visual data is summarized by Field and Chichilnisky who note that: ‘retinal spike trains are significantly more complex than was commonly appreciated, exhibiting surprisingly precise spike timing and highly structured concerted activity in different cells’ [1, p. 2]. They also note that: ‘three-pathway model fails to capture the functional diversity in the LGN. Furthermore, the specificity of connections from LGN to primary visual cortex extends beyond the well-known magnocellular/parvocellular separation [133–136]’ [1, p. 11]. Important challenges of neurophysiological discoveries of the retinal encoding and circuitry are noted to be: ‘identifying the distinct subcircuits that terminate on each RGC type, identifying the diversity of amacrine cell function and its contribution to shaping RGC responses, and identifying how RGCs within and across mosaics interact in communicating visual information to the brain’ [1, p. 11].

The patterns we investigated are black and white, but individual cones are red, green or blue (M, L or S), and RGCs of different types exhibit different colour opponency properties and prevalences and thus different resolutions for different opponent colours. Thus, the contrasts for a black and white image that occur in the retina must be mediated by B–Y or R–G opponents, with the former being primary in mammals [137]. To explore the different spatial frequency characteristics of different RGC types, DeValois and DeValois [138] investigated both chromatic and achromatic versions of the checkerboard illusion. For chromatic and achromatic images *of the same size*, they demonstrated the assimilation in chromatic checkerboards where there was contrast for the achromatic version. They explained that assimilation effect is due to the chromatic system having much lower spatial frequencies compared to the achromatic system. For the Tile Illusions, Westheimer [139] investigate an isoluminant heterochromatic version of one sample Bulge pattern [109] and suggest that the illusion can disappear when the black and white tiles are replaced by isoluminant ones. Although this is not clear in the isoluminant version presented in the article, it can be expected for the above reasons at certain sizes and scales. More psychophysical experiments are needed to clarify the role of colour in Tile Illusions, including testing these illusions in both chromatic and achromatic versions on colour blind people as well as normal subjects. Shapiro [140] developed a quantitative model for the visual response of the cone cells with two separate pathways, one for luminance and chromatic information and one for contrast information. He presented the output of the model for disc-ring stimulus containing two discs of identical chromaticity and luminance, one surrounded by light ring and one surrounded by a dark ring. He showed that the contrast pathway appears to have a faster response compared to the colour pathway (Fig. 5 in [140]). A quantitative model of achromatic colour computation of similar stimulus used in [140] has been investigated by Rudd and Zemach [141] based on a distance-dependent edge integration mechanism. They have shown the outperformance of the edge integration model over the highest luminance rules of Anchoring theory [26].

In conclusion, we have presented a detailed explanation of the extent to which DoG (and LoG) model predicts the Café Wall illusion specifically and measured the degree of tilt quantitatively in each scale utilizing Hough space [48–50]. We have also explored the concept of applying a second-stage processing model for orientation detection of tilt in broader range of Tilt Illusions such as Bulge patterns. The measurement of tilt value should consider both global tilt measurement (overall view of the pattern) and the ‘local focus on tilt’ predicting local tilt percept or the edge displacement. This will highlight a more precise connection of our Tilt Illusion explanation to the perceptual grouping of visual data. We need to estimate the illusion strength and orientation based on a psychophysical assessment of the model prediction, which is a priority in our future study.

In our future work, we intend to add orientation selectivity to the model and make an extension to an implementation of a nonlinear spatial subunits and/or a nCRFs model, inspired by biological findings [95, 142–144]. These extensions can be achieved either by using a summation of Gaussian components at multiple spatial scales, or nCRF implementations. This may be achieved by isotropic filters with three Gaussians such as extended CRF model of Ghosh et al., with classical excitatory and inhibitory surround Gaussians, and a non-classical extended disinhibitory field (surround) [65, 99], or by anisotropic filters with elongated surrounds in different orientations such as the Brightness/Lightness model of Blakeslee and McCourt [31, 67]. The aim will be to explore more on orientation tuning cells and their effects on the perceived brightness, with an attempt to connect the edge map of our model to brightness models, and most importantly to design a bioderived second-stage processing in our model for identifying angles of orientation on detected tilts in the edge map quantitatively instead of the current Hough analysis stage. This extension to the current model facilitates further processing of the revealed tilt in the edge map representation of the patterns. Our intention would be to build our analytical model similar to our visual processing for searching of different visual clues in natural or illusion patterns.

## Appendix

See Tables 1 and 2.

## Footnotes

1 Changizi’s explanation and systematization of Geometrical illusions not only considers perspective clues, but also examines the probable observer’s direction of motion using vanishing point cues, as well as the changes we perceive following the neural lag in the perceived projected size, speed, luminance–contrast, distance and eccentricity of the stimulus which are six correlates of the optic flow, used for illusion prediction and classification.

2 Downloadable from Mathworks—MATLAB central. http://www.mathworks.com/matlabcentral/fileexchange/48419-jetwhite-colours-/content/jetwhite.m.
